# Synthesis and crystal structures of 2-(ferrocenyl­carbon­yl)benzoic acid and 3-ferrocenylphthalide

**DOI:** 10.1107/S2056989020008452

**Published:** 2020-06-30

**Authors:** Uttam R. Pokharel, Jonathan T. Bergeron, Frank R. Fronczek

**Affiliations:** a Department of Chemistry & Physical Sciences, Nicholls State University, Thibodaux, Louisiana 70301, USA; b Department of Chemistry, Louisiana State University, Baton Rouge, Louisiana, 70803, USA

**Keywords:** 3-ferrocenylphthalide, 2-(ferro­cen­yl­carbon­yl)benzoic acid, Zn reduction, crystal structure, 2-carb­oxy­benzyl­ferrocene, redetermination

## Abstract

The title compounds, 2-(ferrocenylcarbon­yl)benzoic acid, [Fe(C_5_H_5_)(C_13_H_9_O_3_)], **1**, and 3-ferrocenylphthalide [systematic name: 3-ferrocenyl-2-benzo­furan-1(3*H*)-one], [Fe(C_5_H_5_)(C_13_H_9_O_2_)], **2**, have been synthesized and structurally characterized by single-crystal X-ray diffraction. The crystal structure of compound **1** was solved recently at room temperature [Qin, Y. (2019 ▸). *CSD Communication* (CCDC deposition number 1912662). CCDC, Cambridge, England]. Here we report a redetermination of its crystal structure at 90 K with improved precision by a factor of about three. The mol­ecular structures of both compounds exhibit a typical sandwich structure. In the crystal packing of compound **1**, each mol­ecule engages in inter­molecular hydrogen bonding, forming a centrosymmetric dimer with graph-set notation *R* ^2^_2_ (8) and an O⋯O distance of 2.6073 (15) Å. There are weak C—H⋯O and C—H⋯π inter­actions in the crystal packing of compound **2**. The phthalide moiety in **2** is oriented roughly perpendicular to the ferrocene backbone, with a dihedral angle of 77.4 (2)°.

## Abstract 2

2-Ferrocenyl­carbonyl­benzoic acid (C_18_H_14_FeO_3_) was synthesized from the Friedel–Crafts acyl­ation of ferrocene with phthalic anhydride, and the product was reduced to 3-ferrocenylphthalide (C_18_H_14_FeO_2_) using Zn(Cu) in aqueous sodium hydroxide. Both compounds were characterized using IR, NMR, and single-crystal X-ray analysis.

## Chemical context   

Our research group has been inter­ested in developing methodologies to synthesize metallocene-fused quinones as synthetic precursors of π-extended metallocenes. These are of inter­est because an integration of the redox-active metal center with the polycyclic aromatic hydro­carbons could alter their properties for organic semiconducting applications (Anthony, 2006 ▸). Previously, we synthesized metallocene-fused quinones *via* the double Friedel–Crafts acyl­ation reaction between 1′,2′,3′,4′,5′-penta­methyl­ruthenocene-1,2-diacyl chloride with organic aromatics (Pokharel *et al.*, 2011 ▸). Later, we realized that switching the functionality of two reaction partners allows us to obtain quinones in a much simpler synthetic scheme. Ferrocene being a close analog of ruthenocene, we decided to pursue the synthesis of ferrocene-fused quinones (Nesmeyanov *et al.*, 1966 ▸; Pokharel, 2012 ▸), starting from ferrocene itself as the aromatic reagent. As the first step of this synthetic route, we prepared 2-ferrocenylcarbonyl benzoic acid, **1**, following a previously reported procedure (Shen *et al.*, 2012 ▸; Xu *et al.*, 2017 ▸). The published procedure uses di­chloro­methane as the reaction solvent. However, using this solvent, we obtained consistently low reaction yields. On switching to di­chloro­ethane from di­chloro­methane, the yield of the reaction was improved from 13% to a more satisfactory 51% even at room temperature, possibly due to higher solubility of the reaction mixture. The crystal structure of the complex has been reported at room temperature (Qin, 2019 ▸). Our redetermination of its crystal structure at 90 K has improved the precision by a factor of about three.
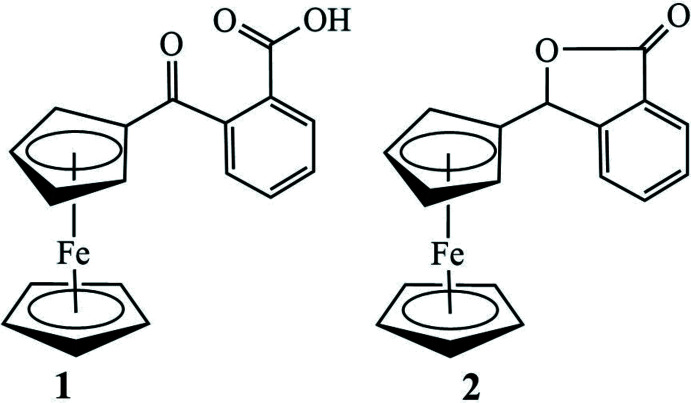


**Figure 1:**
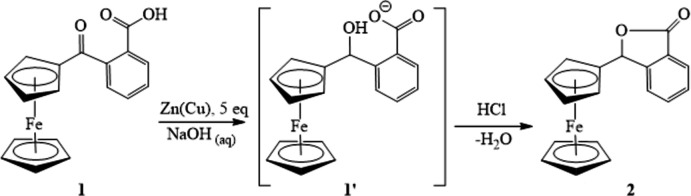
The synthetic scheme to the formation of unexpected title compound **2** from the title compound **1** with proposed inter­mediate.

With an easy route towards 2-ferrocenylcarbonyl benzoic acid, **1**, at hand, we investigated the reduction of its keto group to methyl­ene using a large excess of zinc powder (*ca* 48 equivalents) with aqueous sodium hydroxide as the solvent (Lee & Harvey, 1986 ▸). Under these reaction conditions, we were able to reduce complex **1** to 2-carb­oxy­benzyl­ferrocene in 89% yield (Pokharel, 2012 ▸). Following this successful transformation, we investigated the reaction outcome in the presence of a smaller amount (5 equivalents) of Zn. Under these reaction conditions, the reaction mixture changed color from red to light orange. However, on acidification, the reaction yielded the title compound **2** in a 77% yield. We assume that the limited amount of zinc leads to incomplete reduction of the ketone to a secondary alcohol, **1′** (Fig. 1 ▸), similar to the reduction of aryl ketones reported by Zhang and co-workers (Zhang *et al.*, 2007 ▸). Upon acidification during reaction workup, alcohol **1′** undergoes solvolysis to give the carbocation, which is electronically stabilized by the ferrocenyl group (Goodman *et al.*, 2019 ▸). The nucleophilic attack of the carb­oxy­lic O atom leads to the formation of the cyclic lactone, **2**. Although the title compound **2** was reported long ago as a major product from the reaction of 3,3′-diferrocenyl-3,3′-diphthalide with KOH (Nesmeyanov *et al.*, 1961 ▸) and as a byproduct from the polycondensation reaction of ferrocene with *o*-carb­oxy­benzaldehyde (Neuse & Koda, 1966 ▸), to our knowledge, this is the first report of the conversion of keto carb­oxy­lic acid, **1**, to cyclic lactone **2** in a reasonably high yield. Here we report the synthesis, spectroscopic characterization, and single-crystal X-ray analysis of the title compounds **1** and **2**.

## Structural commentary   

**Figure 2:**
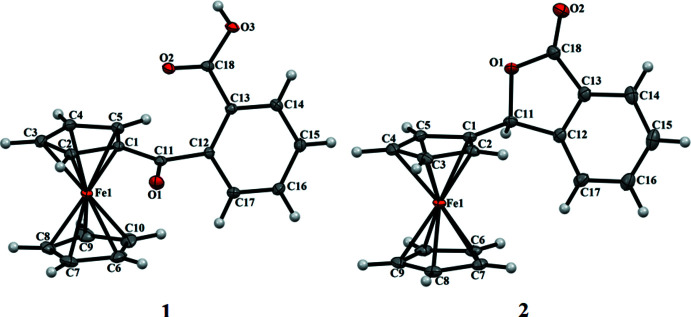
Mol­ecular structure of the title compounds **1** and **2** showing the atom-numbering schemes. Displacement ellipsoids are drawn at the 50% probability level.

A view of the mol­ecular structures of the title compounds **1** and **2**, with their atom labeling, is shown in Fig. 2 ▸. The ferrocenyl moieties adopt typical sandwich structures with Fe—C distances in the range 2.0287 (17)–2.0498 (15) Å in compound **1** and of 2.032 (2)–2.055 (2) Å in **2**. In both structures, the Fe—C bond lengths towards the substituted carbon are shorter [Fe—C1 2.031 (1) Å in **1**; 2.032 (2) Å in **2**] than the remaining Fe—C bond lengths, similar to literature reports (Pérez *et al.*, 2015 ▸; Wu *et al.*, 2011 ▸). The C—C distances within the Cp rings fall in the range 1.412 (2)–1.429 (2) Å in compound **1** and 1.414 (3)–1.431 (3) Å in **2**. Similar to its carboxyl­ate salts (Li *et al.*, 2003 ▸; Li, Li *et al.*, 2008 ▸; Li, Liu *et al.*, 2008 ▸; Xu *et al.*, 2016 ▸), the two Cp rings of the ferrocene residue in complex **1** are close to an eclipsed conformation (mean of five C—*Cg*—*Cg*—C torsion angles = 12.68°; *Cg* is the centroid of the respective cyclo­penta­dienyl ring). The analogous angle in complex **2** is 3.31°. The Cp rings are essentially parallel in both complexes, making a dihedral angle of 2.45 (12)° in compound **1** and 1.14 (10)° in **2**. The Fe⋯*Cg* distances in both compounds are in a similar range [substituted and unsubstituted Cp in **1**: 1.6436 (7) and 1.6458 (7) Å; **2**: 1.6455 (10) and 1.6510 (10) Å, respectively]. The *Cg*—Fe—*Cg* angle in both structures is *ca* 178°. The carbonyl carbon, C11 in compound **1** bends toward the iron center with a distance of 0.163 (3) from the least-squares plane of the substituted Cp while the corresponding C11 atom in compound **2** bends slightly outward with a distance of 0.117 (4) Å from the plane of Cp. Similar bending can be seen in the *N*-imidazolyl derivative of compound **2** (Simenel *et al.*, 2008 ▸). The carbonyl carbon in compound **1** lies roughly in the same plane as the substituted Cp with a torsional angle C2—C1—C11—O1 of 2.9 (2)°. The phenyl ring in compound **1** is twisted away from the plane of the carbonyl (C=O) plane with a torsional angle O1—C11—C12—C13 of −112.41 (16)°. The aromatic ring of the phthalide moiety in compound **2** bends away from ferrocene and orients roughly perpendicular to the ferrocene backbone. The nine-atom phthalide plane of compound **2** inclines with the substituted Cp at a dihedral angle of 77.31 (7)°. This mol­ecule contains a single asymmetric center at the C11 position in this racemic structure.

## Supra­molecular features   

**Figure 3:**
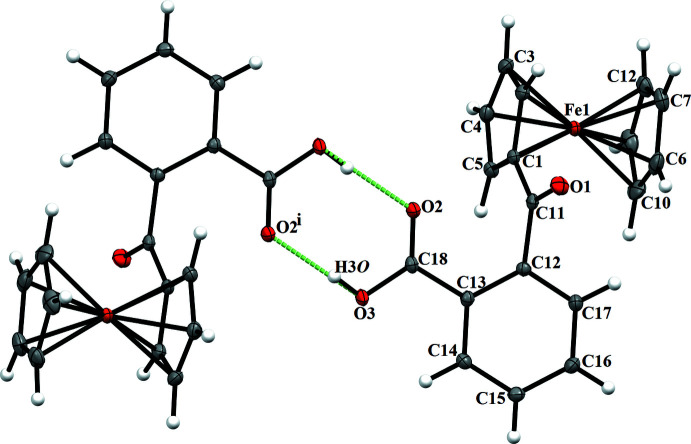
The hydrogen-bonded dimer of title compound **1**. Unlabeled atoms are related to their labeled counterparts by a crystallographic inversion center [Symmetry code: (i) −*x*, −*y*, 1 − *z*]. Displacement ellipsoids are drawn at the 50% probability level.

**Figure 4:**
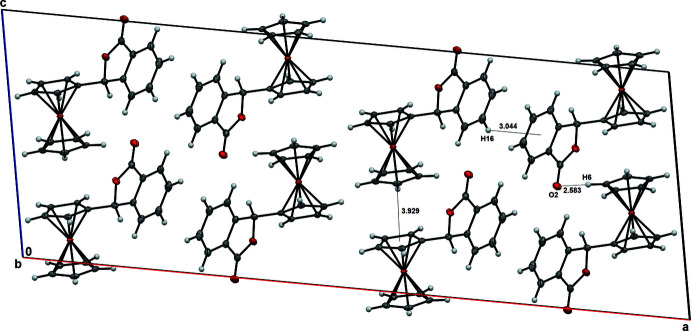
The crystal packing of title compound **2**, viewed along the *b* axis. Displacement ellipsoids are drawn at the 50% probability level.

The mol­ecules in compound **1** are associated *via* classical hydrogen-bonding inter­actions between the carb­oxy­lic OH group of one mol­ecule with the carbonyl oxygen of an adjacent mol­ecule. The carb­oxy­lic acid groups are related *via* a crystallographic inversion center to form hydrogen bonds [O3—H3*O*⋯O2^i^ [symmetry code: (i) −*x*, −*y*, 1 − *z*] with an 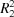 (8) dimer (Etter *et al.*, 1990 ▸) motif (Table 1 ▸ and Fig. 3 ▸). This centrosymmetric pairwise hydrogen-bonding dimer formation results in short hydrogen-bond distances of 2.6073 (15). In the crystal packing of title compound **2** (Fig. 4 ▸), the unsubstituted Cp orients towards the substituted Cp of a mol­ecule at *x*, 1 − *y*, *z* − 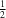 with a *Cg*⋯*Cg* separation of 3.929 (1) Å. There is a weak hydrogen-bonding inter­action between the carbonyl oxygen O2 of the phthalide ring, and hydrogen H6 of the unsubstituted Cp with an H6⋯O2 (*x*, 2 − *y*, *z* − 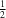) distance of 2.58 Å (Table 2 ▸). The phthalide moieties in the two mol­ecules are oriented at an angle of 73.49° and exhibit a weak C—H⋯π inter­action as evidenced by the distance of 3.044 Å between H16 and the centroid of the aromatic ring of a phthalide moiety at 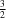 − *x*, *y* − 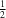, 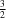 − *z*.

## Database survey   

The structure of title compound **1** (CSD refcode JOJGOH) at room temperature has been recently reported as a CSD Communication (Qin, 2019 ▸) but no details of the mol­ecular or crystal structure were provided. Various salts of this carb­oxy­lic acid: sodium (LULSAN; Li, Liu *et al.*, 2008 ▸), magnesium (ADULUJ; Xu *et al.*, 2016 ▸), barium (ECIVIY; Xu *et al.*, 2017 ▸), zinc (CIXNED; Li, Li *et al.*, 2008 ▸), cadmium (IKAZID), zinc (IKAZEZ), and lead(II) (IKAZOJ) (Li *et al.*, 2003 ▸) have been reported. The structure of a compound analogous to the title compound **2** but with an *N*-imidazolyl group at C11 has also been reported (VIYTIH; Simenel *et al.*, 2008 ▸). That structure has a disorder of the ferrocenyl substituent involving both eclipsed and staggered conformations.

## Synthesis and crystallization   

**2-Ferrocenylcarbonyl benzoic acid (1).** To a stirred solution of phthalic anhydride (16.00 g, 0.108 mol) and AlCl_3_ (14.4 g, 0.108 mol) in di­chloro­ethane (60 mL), ferrocene (10.00 g, 0.053 mol) in di­chloro­ethane (65 mL) was added dropwise. The reaction mixture was stirred for 2 h at room temperature, and the mixture poured into ice-cold water (400 mL). The product was extracted with di­chloro­methane (2 × 250 mL). The organic phase was collected and again extracted with 2 *M* NaOH (3 × 100 mL). The combined aqueous phase was acidified with conc. HCl until the pH dropped into the 2–3 range. The precipitate was filtered off, washed with water (200 mL), and dried under vacuum to give **1** (9.20 g, 51%) as a red–brown crystalline solid. Crystals suitable for single-crystal X-ray diffraction were grown by slow evaporation, at ambient temperature, of a solution in a mixture of hexane and diethyl ether. M.p. 457–459 K [Lit. 459 K (Nesmeyanov *et al.*, 1961 ▸)]. IR (ATR, cm^−1^): 1652 (C=O), 1688 (C=O), 2600–3200 (OH). ^1^H NMR (400 MHz, acetone-*d*_6_, ppm): δ 4.22 (*s*, 5H, Cp) 4.53 (*br*, 4H, Cp), 7.62–7.66 (*m*, 1H, Ar), 7.72–7.79 (*m*, 2H, Ar), 7.98 (*dd*, 1H, ^3^ *J* = 7.6 Hz, ^4^ *J* = 0.8 Hz, Ar). ^13^C NMR (100 MHz, acetone-*d*_6_, ppm): δ 70.7, 70.8, 72.9, 81.4 (Cp), 128.9, 130.3, 130.6, 130.7, 133.0, 143.8 (Ar), 167.7 (*C*OOH), 200.1 (*C*O).

**3-Ferrocenylphthalide (2).** In a 250 mL Schlenk flask, zinc powder (5.0 g, 0.076 mol) was activated by stirring it in a solution of CuSO_4_ (0.17 g, 0.0011 mol) in DI water (15 mL) for 10 minutes. The solution was deca­nted, and the residue was washed with water (50 mL). To the activated zinc, keto-acid **1** (5.0 g, 0.015 mol) in NaOH solution (4.80 g in 30 mL of water) was added. The reaction mixture was allowed to reflux for 5 h, and then cooled to room temperature. The reaction mixture was filtered, and the filtrate acidified with conc. HCl. The resulting precipitate was collected, washed with water, and dried to give a viscous mass. The crude product was redissolved in di­chloro­methane (100 mL) and the acidic impurities extracted with 1 *M* NaOH (2 × 10 mL). The organic layer was collected, dried with anhydrous MgSO_4_, filtered, and the filtrate evaporated to dryness to give the title compound **2** (3.65 g, 77%) as a pale-yellow solid. Crystals suitable for single-crystal X-ray diffraction were grown by slow evaporation, at ambient temperature, of a solution in a mixture of hexane and diethyl ether. M..p: 410–411 K. IR (ATR, cm^−1^): 1760 (*s*); 1286 (*s*); 1068 (*s*). ^1^H NMR (400 MHz; acetone-*d*_6_; ppm): δ 4.14 (*br*, 1H, Cp), 4.20 (*s*, 5H, Cp), 4.21 (*m*, 1H, Cp), 4.25 (*br*, 1H, Cp), 4.30 (*br*, 1H, Cp), 6.44 (*s*, 1H, C*H*), 7.63 (*br*, 1H, Ar), 7.78–7.84 (*m*, 3H, Ar). ^13^C NMR (100 MHz, acetone-*d*_6_, ppm): δ 66.7, 66.9, 68.2, 68.9, 79.6, 85.2, 123.4, 125.0, 126.1, 129.4, 134.1, 149.5, 169.5.

## Refinement   

Crystal data, data collection and structure refinement details are summarized in Table 3 ▸. All H atoms were located in difference maps and then treated as riding in geometrically idealized positions with C—H distances of 1.00 Å (0.95 Å phen­yl) and with *U*_iso_(H) =1.2*U*_eq_ for the attached C atom. The coordinates of the OH hydrogen atom in **1** were refined with the O—H distance restrained to 0.88 (2) Å, and its *U*_iso_ value was assigned as 1.5*U*_eq_ of the O atom.

## Supplementary Material

Crystal structure: contains datablock(s) 1, 2. DOI: 10.1107/S2056989020008452/zl2783sup1.cif

Structure factors: contains datablock(s) 1. DOI: 10.1107/S2056989020008452/zl27831sup2.hkl

Supporting information file. DOI: 10.1107/S2056989020008452/zl27831sup4.cdx

Structure factors: contains datablock(s) 2. DOI: 10.1107/S2056989020008452/zl27832sup3.hkl

CCDC references: 2011817, 2011818

Additional supporting information:  crystallographic information; 3D view; checkCIF report

## Figures and Tables

**Table 1:** Hydrogen-bond geometry (Å, °) for **1** [Chem scheme1]

*D*—H⋯*A*	*D*—H	H⋯*A*	*D*⋯*A*	*D*—H⋯*A*
O3—H3*O*⋯O2^i^	0.82 (2)	1.79 (2)	2.6073 (15)	174 (2)

Symmetry code: (i) 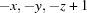.

**Table 2:** Hydrogen-bond geometry (Å, °) for **2** [Chem scheme1]

*D*—H⋯*A*	*D*—H	H⋯*A*	*D*⋯*A*	*D*—H⋯*A*
C6—H6⋯O2^i^	1.00	2.58	3.470 (3)	148

Symmetry code: (i) 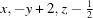.

**Table 3:** Experimental details

	**1**	**2**
Crystal data		
Chemical formula	[Fe(C_5_H_5_)(C_13_H_9_O_3_)]	[Fe(C_5_H_5_)(C_13_H_9_O_2_)]
*M*_r_	334.14	318.14
Crystal system, space group	Monoclinic, *P*2_1_/*c*	Monoclinic, *C*2/*c*
Temperature (K)	110	110
*a*, *b*, *c* (Å)	17.1332 (13), 7.4478 (5), 11.0345 (8)	35.4613 (11), 5.6873 (2), 13.1523 (4)
β (°)	105.758 (4)	100.2019 (16)
*V* (Å^3^)	1355.13 (17)	2610.61 (15)
*Z*	4	8
Radiation type	Mo *K*α	Mo *K*α
μ (mm^−1^)	1.12	1.16
Crystal size (mm)	0.25 × 0.12 × 0.05	0.42 × 0.38 × 0.03

Data collection		
Diffractometer	Bruker Kappa APEXII DUO CCD	Bruker Kappa APEXII DUO CCD
Absorption correction	Multi-scan (*SADABS*; Krause *et al.*, 2015 ▸)	Multi-scan (*SADABS*; Krause *et al.*, 2015 ▸)
*T*_min_, *T*_max_	0.891, 0.946	0.826, 0.966
No. of measured, independent and observed [*I* > 2σ(*I*)] reflections	25434, 4737, 3834	18463, 4536, 3996
*R*_int_	0.037	0.028
(sin θ/λ)_max_ (Å^−1^)	0.748	0.748

Refinement		
*R*[*F* ^2^ > 2σ(*F* ^2^)], *wR*(*F* ^2^), *S*	0.033, 0.082, 1.04	0.047, 0.116, 1.18
No. of reflections	4737	4536
No. of parameters	202	190
No. of restraints	1	0
H-atom treatment	H atoms treated by a mixture of independent and constrained refinement	H-atom parameters constrained
Δρ_max_, Δρ_min_ (e Å^−3^)	0.55, −0.28	0.91, −0.49

Computer programs: *APEX3* and *SAINT* (Bruker, 2016 ▸), *SHELXS97* (Sheldrick, 2008 ▸), *SHELXL2014/7* (Sheldrick, 2015 ▸), *Mercury* (Macrae *et al.*, 2020 ▸) and *publCIF* (Westrip, 2010 ▸).

## supplementary crystallographic information

### 2-(Ferrocenylcarbonyl)benzoic acid (1) Crystal data

**Table:**

[Fe(C_5_H_5_)(C_13_H_9_O_3_)]	*F*(000) = 688
*M**_r_* = 334.14	*D*_x_ = 1.638 Mg m^−^^3^
Monoclinic, *P*2_1_/*c*	Mo *K*α radiation, λ = 0.71073 Å
*a* = 17.1332 (13) Å	Cell parameters from 7843 reflections
*b* = 7.4478 (5) Å	θ = 2.5–32.0°
*c* = 11.0345 (8) Å	µ = 1.12 mm^−^^1^
β = 105.758 (4)°	*T* = 110 K
*V* = 1355.13 (17) Å^3^	Plate, yellow-orange
*Z* = 4	0.25 × 0.12 × 0.05 mm

### 2-(Ferrocenylcarbonyl)benzoic acid (1) Data collection

**Table:**

Bruker Kappa APEXII DUO CCD diffractometer	4737 independent reflections
Radiation source: fine-focus sealed tube	3834 reflections with *I* > 2σ(*I*)
TRIUMPH curved graphite monochromator	*R*_int_ = 0.037
φ and ω scans	θ_max_ = 32.1°, θ_min_ = 1.2°
Absorption correction: multi-scan (SADABS; Krause *et al.*, 2015)	*h* = −25→25
*T*_min_ = 0.891, *T*_max_ = 0.946	*k* = −11→11
25434 measured reflections	*l* = −16→16

### 2-(Ferrocenylcarbonyl)benzoic acid (1) Refinement

**Table:**

Refinement on *F*^2^	Primary atom site location: structure-invariant direct methods
Least-squares matrix: full	Secondary atom site location: difference Fourier map
*R*[*F*^2^ > 2σ(*F*^2^)] = 0.033	Hydrogen site location: mixed
*wR*(*F*^2^) = 0.082	H atoms treated by a mixture of independent and constrained refinement
*S* = 1.04	*w* = 1/[σ^2^(*F*_o_^2^) + (0.0391*P*)^2^ + 0.6785*P*] where *P* = (*F*_o_^2^ + 2*F*_c_^2^)/3
4737 reflections	(Δ/σ)_max_ < 0.001
202 parameters	Δρ_max_ = 0.55 e Å^−^^3^
1 restraint	Δρ_min_ = −0.28 e Å^−^^3^

### 2-(Ferrocenylcarbonyl)benzoic acid (1) Special details

**Table:**

Geometry. All esds (except the esd in the dihedral angle between two l.s. planes) are estimated using the full covariance matrix. The cell esds are taken into account individually in the estimation of esds in distances, angles and torsion angles; correlations between esds in cell parameters are only used when they are defined by crystal symmetry. An approximate (isotropic) treatment of cell esds is used for estimating esds involving l.s. planes.

### 2-(Ferrocenylcarbonyl)benzoic acid (1) Fractional atomic coordinates and isotropic or equivalent isotropic displacement parameters (Å^2^)

**Table:**

	*x*	*y*	*z*	*U*_iso_*/*U*_eq_	
Fe1	0.35842 (2)	0.21980 (3)	0.79339 (2)	0.01110 (6)	
O1	0.16447 (6)	0.39240 (15)	0.82421 (10)	0.0166 (2)	
O2	0.07156 (6)	0.12073 (14)	0.59281 (9)	0.0148 (2)	
O3	0.00212 (6)	0.18223 (15)	0.39475 (10)	0.0156 (2)	
H3O	−0.0206 (12)	0.089 (2)	0.4037 (19)	0.023*	
C1	0.23630 (8)	0.18631 (19)	0.73350 (13)	0.0112 (2)	
C2	0.26963 (8)	0.0784 (2)	0.84282 (13)	0.0139 (3)	
H2	0.2556	0.0867	0.9249	0.017*	
C3	0.32643 (8)	−0.0411 (2)	0.81410 (14)	0.0162 (3)	
H3A	0.3600	−0.1311	0.8730	0.019*	
C4	0.32878 (8)	−0.0089 (2)	0.68811 (14)	0.0155 (3)	
H4	0.3640	−0.0727	0.6431	0.019*	
C5	0.27296 (8)	0.1310 (2)	0.63776 (13)	0.0133 (3)	
H5	0.2618	0.1824	0.5510	0.016*	
C6	0.37747 (9)	0.4747 (2)	0.85968 (17)	0.0226 (3)	
H6	0.3348	0.5605	0.8700	0.027*	
C7	0.42094 (9)	0.3531 (2)	0.95154 (15)	0.0201 (3)	
H7	0.4140	0.3368	1.0380	0.024*	
C8	0.47552 (9)	0.2566 (2)	0.89958 (15)	0.0194 (3)	
H8	0.5135	0.1600	0.9429	0.023*	
C9	0.46628 (9)	0.3191 (2)	0.77533 (16)	0.0232 (3)	
H9	0.4966	0.2752	0.7155	0.028*	
C10	0.40535 (10)	0.4548 (2)	0.75049 (17)	0.0252 (4)	
H10	0.3856	0.5238	0.6702	0.030*	
C11	0.18211 (8)	0.3415 (2)	0.73014 (13)	0.0113 (2)	
C12	0.15537 (7)	0.45069 (19)	0.61065 (12)	0.0105 (2)	
C13	0.09795 (7)	0.39345 (19)	0.50127 (13)	0.0109 (2)	
C14	0.07597 (8)	0.5053 (2)	0.39646 (13)	0.0127 (3)	
H14	0.0369	0.4658	0.3226	0.015*	
C15	0.11056 (8)	0.6736 (2)	0.39890 (13)	0.0143 (3)	
H15	0.0959	0.7489	0.3267	0.017*	
C16	0.16672 (8)	0.7316 (2)	0.50722 (14)	0.0153 (3)	
H16	0.1905	0.8472	0.5096	0.018*	
C17	0.18832 (8)	0.6210 (2)	0.61246 (13)	0.0141 (3)	
H17	0.2263	0.6628	0.6868	0.017*	
C18	0.05658 (7)	0.2189 (2)	0.50034 (13)	0.0113 (2)	

### 2-(Ferrocenylcarbonyl)benzoic acid (1) Atomic displacement parameters (Å^2^)

**Table:**

	*U*^11^	*U*^22^	*U*^33^	*U*^12^	*U*^13^	*U*^23^
Fe1	0.00902 (9)	0.01024 (10)	0.01257 (10)	−0.00193 (7)	0.00041 (6)	−0.00021 (7)
O1	0.0193 (5)	0.0185 (5)	0.0126 (5)	0.0008 (4)	0.0055 (4)	−0.0015 (4)
O2	0.0141 (4)	0.0142 (5)	0.0132 (5)	−0.0044 (4)	−0.0012 (3)	0.0016 (4)
O3	0.0164 (4)	0.0157 (5)	0.0118 (5)	−0.0078 (4)	−0.0012 (4)	0.0005 (4)
C1	0.0097 (5)	0.0104 (6)	0.0120 (6)	−0.0019 (4)	0.0003 (4)	0.0001 (5)
C2	0.0131 (5)	0.0125 (7)	0.0145 (6)	−0.0031 (5)	0.0009 (5)	0.0014 (5)
C3	0.0149 (6)	0.0106 (7)	0.0203 (7)	−0.0011 (5)	0.0000 (5)	0.0023 (5)
C4	0.0142 (6)	0.0124 (7)	0.0181 (7)	−0.0002 (5)	0.0013 (5)	−0.0035 (5)
C5	0.0124 (5)	0.0130 (7)	0.0126 (6)	−0.0007 (5)	0.0002 (5)	−0.0009 (5)
C6	0.0170 (6)	0.0136 (7)	0.0336 (9)	−0.0042 (5)	0.0005 (6)	−0.0048 (6)
C7	0.0157 (6)	0.0235 (8)	0.0184 (7)	−0.0054 (6)	0.0003 (5)	−0.0070 (6)
C8	0.0109 (5)	0.0215 (8)	0.0222 (7)	−0.0019 (5)	−0.0018 (5)	−0.0032 (6)
C9	0.0142 (6)	0.0303 (9)	0.0252 (8)	−0.0091 (6)	0.0053 (6)	−0.0026 (7)
C10	0.0218 (7)	0.0196 (8)	0.0302 (9)	−0.0108 (6)	0.0002 (6)	0.0072 (7)
C11	0.0094 (5)	0.0114 (6)	0.0122 (6)	−0.0033 (4)	0.0013 (4)	−0.0011 (5)
C12	0.0093 (5)	0.0106 (6)	0.0113 (6)	0.0007 (4)	0.0022 (4)	−0.0004 (5)
C13	0.0095 (5)	0.0108 (6)	0.0122 (6)	−0.0006 (4)	0.0025 (4)	−0.0012 (5)
C14	0.0124 (5)	0.0143 (7)	0.0105 (6)	−0.0003 (5)	0.0013 (4)	−0.0004 (5)
C15	0.0146 (6)	0.0136 (7)	0.0140 (6)	0.0006 (5)	0.0027 (5)	0.0030 (5)
C16	0.0160 (6)	0.0106 (6)	0.0180 (7)	−0.0016 (5)	0.0024 (5)	0.0013 (5)
C17	0.0150 (6)	0.0119 (7)	0.0133 (6)	−0.0014 (5)	0.0002 (5)	−0.0016 (5)
C18	0.0091 (5)	0.0127 (6)	0.0118 (6)	−0.0015 (5)	0.0022 (4)	−0.0022 (5)

### 2-(Ferrocenylcarbonyl)benzoic acid (1) Geometric parameters (Å, º)

**Table:**

Fe1—C6	2.0287 (17)	C5—H5	1.0000
Fe1—C1	2.0311 (13)	C6—C7	1.412 (2)
Fe1—C10	2.0351 (16)	C6—C10	1.419 (3)
Fe1—C7	2.0403 (15)	C6—H6	1.0000
Fe1—C5	2.0409 (13)	C7—C8	1.418 (2)
Fe1—C2	2.0424 (14)	C7—H7	1.0000
Fe1—C4	2.0468 (15)	C8—C9	1.415 (2)
Fe1—C8	2.0485 (14)	C8—H8	1.0000
Fe1—C3	2.0485 (15)	C9—C10	1.425 (2)
Fe1—C9	2.0498 (15)	C9—H9	1.0000
O1—C11	1.2179 (17)	C10—H10	1.0000
O2—C18	1.2243 (17)	C11—C12	1.5107 (19)
O3—C18	1.3085 (16)	C12—C17	1.387 (2)
O3—H3O	0.816 (15)	C12—C13	1.4005 (18)
C1—C5	1.429 (2)	C13—C14	1.3918 (19)
C1—C2	1.4328 (19)	C13—C18	1.4797 (19)
C1—C11	1.476 (2)	C14—C15	1.383 (2)
C2—C3	1.417 (2)	C14—H14	0.9500
C2—H2	1.0000	C15—C16	1.384 (2)
C3—C4	1.422 (2)	C15—H15	0.9500
C3—H3A	1.0000	C16—C17	1.389 (2)
C4—C5	1.421 (2)	C16—H16	0.9500
C4—H4	1.0000	C17—H17	0.9500
			
C6—Fe1—C1	106.43 (6)	C5—C4—Fe1	69.43 (8)
C6—Fe1—C10	40.88 (7)	C3—C4—Fe1	69.75 (8)
C1—Fe1—C10	117.84 (6)	C5—C4—H4	125.9
C6—Fe1—C7	40.60 (7)	C3—C4—H4	125.9
C1—Fe1—C7	126.38 (6)	Fe1—C4—H4	125.9
C10—Fe1—C7	68.39 (7)	C4—C5—C1	107.86 (13)
C6—Fe1—C5	127.97 (6)	C4—C5—Fe1	69.88 (8)
C1—Fe1—C5	41.08 (5)	C1—C5—Fe1	69.10 (8)
C10—Fe1—C5	108.76 (6)	C4—C5—H5	126.1
C7—Fe1—C5	165.35 (6)	C1—C5—H5	126.1
C6—Fe1—C2	116.41 (7)	Fe1—C5—H5	126.1
C1—Fe1—C2	41.19 (5)	C7—C6—C10	108.02 (15)
C10—Fe1—C2	151.12 (7)	C7—C6—Fe1	70.14 (9)
C7—Fe1—C2	106.17 (6)	C10—C6—Fe1	69.80 (10)
C5—Fe1—C2	68.98 (6)	C7—C6—H6	126.0
C6—Fe1—C4	166.99 (7)	C10—C6—H6	126.0
C1—Fe1—C4	68.79 (6)	Fe1—C6—H6	126.0
C10—Fe1—C4	129.55 (7)	C6—C7—C8	108.18 (15)
C7—Fe1—C4	151.90 (6)	C6—C7—Fe1	69.26 (9)
C5—Fe1—C4	40.70 (6)	C8—C7—Fe1	70.02 (9)
C2—Fe1—C4	68.56 (6)	C6—C7—H7	125.9
C6—Fe1—C8	68.40 (6)	C8—C7—H7	125.9
C1—Fe1—C8	164.76 (6)	Fe1—C7—H7	125.9
C10—Fe1—C8	68.32 (7)	C9—C8—C7	108.21 (15)
C7—Fe1—C8	40.59 (6)	C9—C8—Fe1	69.85 (8)
C5—Fe1—C8	153.14 (6)	C7—C8—Fe1	69.40 (8)
C2—Fe1—C8	126.94 (6)	C9—C8—H8	125.9
C4—Fe1—C8	119.31 (6)	C7—C8—H8	125.9
C6—Fe1—C3	150.36 (7)	Fe1—C8—H8	125.9
C1—Fe1—C3	68.69 (6)	C8—C9—C10	107.64 (15)
C10—Fe1—C3	167.54 (7)	C8—C9—Fe1	69.74 (9)
C7—Fe1—C3	117.40 (7)	C10—C9—Fe1	69.02 (9)
C5—Fe1—C3	68.52 (6)	C8—C9—H9	126.2
C2—Fe1—C3	40.52 (6)	C10—C9—H9	126.2
C4—Fe1—C3	40.64 (6)	Fe1—C9—H9	126.2
C8—Fe1—C3	108.25 (6)	C6—C10—C9	107.96 (15)
C6—Fe1—C9	68.68 (7)	C6—C10—Fe1	69.32 (9)
C1—Fe1—C9	152.73 (6)	C9—C10—Fe1	70.14 (9)
C10—Fe1—C9	40.84 (7)	C6—C10—H10	126.0
C7—Fe1—C9	68.28 (7)	C9—C10—H10	126.0
C5—Fe1—C9	119.66 (6)	Fe1—C10—H10	126.0
C2—Fe1—C9	165.59 (6)	O1—C11—C1	121.49 (13)
C4—Fe1—C9	109.66 (7)	O1—C11—C12	119.31 (13)
C8—Fe1—C9	40.41 (7)	C1—C11—C12	118.88 (12)
C3—Fe1—C9	128.95 (7)	C17—C12—C13	118.61 (13)
C18—O3—H3O	108.5 (14)	C17—C12—C11	117.03 (12)
C5—C1—C2	107.82 (12)	C13—C12—C11	124.32 (12)
C5—C1—C11	127.38 (13)	C14—C13—C12	120.14 (13)
C2—C1—C11	124.35 (12)	C14—C13—C18	119.96 (12)
C5—C1—Fe1	69.83 (7)	C12—C13—C18	119.77 (12)
C2—C1—Fe1	69.83 (7)	C15—C14—C13	120.56 (12)
C11—C1—Fe1	119.82 (9)	C15—C14—H14	119.7
C3—C2—C1	107.75 (12)	C13—C14—H14	119.7
C3—C2—Fe1	69.97 (8)	C14—C15—C16	119.54 (13)
C1—C2—Fe1	68.99 (8)	C14—C15—H15	120.2
C3—C2—H2	126.1	C16—C15—H15	120.2
C1—C2—H2	126.1	C15—C16—C17	120.11 (14)
Fe1—C2—H2	126.1	C15—C16—H16	119.9
C2—C3—C4	108.45 (13)	C17—C16—H16	119.9
C2—C3—Fe1	69.50 (8)	C12—C17—C16	121.02 (13)
C4—C3—Fe1	69.62 (9)	C12—C17—H17	119.5
C2—C3—H3A	125.8	C16—C17—H17	119.5
C4—C3—H3A	125.8	O2—C18—O3	123.72 (13)
Fe1—C3—H3A	125.8	O2—C18—C13	121.82 (12)
C5—C4—C3	108.11 (13)	O3—C18—C13	114.44 (12)
			
C5—C1—C2—C3	0.28 (15)	C7—C6—C10—Fe1	59.98 (11)
C11—C1—C2—C3	−172.45 (12)	C8—C9—C10—C6	−0.06 (18)
Fe1—C1—C2—C3	−59.46 (10)	Fe1—C9—C10—C6	59.21 (11)
C5—C1—C2—Fe1	59.73 (9)	C8—C9—C10—Fe1	−59.27 (11)
C11—C1—C2—Fe1	−112.99 (13)	C5—C1—C11—O1	−168.41 (13)
C1—C2—C3—C4	−0.05 (16)	C2—C1—C11—O1	2.9 (2)
Fe1—C2—C3—C4	−58.89 (10)	Fe1—C1—C11—O1	−81.98 (15)
C1—C2—C3—Fe1	58.84 (9)	C5—C1—C11—C12	5.0 (2)
C2—C3—C4—C5	−0.20 (16)	C2—C1—C11—C12	176.29 (12)
Fe1—C3—C4—C5	−59.02 (10)	Fe1—C1—C11—C12	91.44 (13)
C2—C3—C4—Fe1	58.82 (10)	O1—C11—C12—C17	65.23 (17)
C3—C4—C5—C1	0.37 (16)	C1—C11—C12—C17	−108.34 (15)
Fe1—C4—C5—C1	−58.85 (9)	O1—C11—C12—C13	−112.41 (16)
C3—C4—C5—Fe1	59.22 (10)	C1—C11—C12—C13	74.02 (17)
C2—C1—C5—C4	−0.40 (15)	C17—C12—C13—C14	1.08 (19)
C11—C1—C5—C4	172.04 (13)	C11—C12—C13—C14	178.69 (12)
Fe1—C1—C5—C4	59.33 (9)	C17—C12—C13—C18	−174.74 (12)
C2—C1—C5—Fe1	−59.73 (9)	C11—C12—C13—C18	2.9 (2)
C11—C1—C5—Fe1	112.71 (13)	C12—C13—C14—C15	0.2 (2)
C10—C6—C7—C8	−0.36 (17)	C18—C13—C14—C15	175.97 (12)
Fe1—C6—C7—C8	59.41 (11)	C13—C14—C15—C16	−0.8 (2)
C10—C6—C7—Fe1	−59.77 (11)	C14—C15—C16—C17	0.2 (2)
C6—C7—C8—C9	0.33 (17)	C13—C12—C17—C16	−1.7 (2)
Fe1—C7—C8—C9	59.26 (11)	C11—C12—C17—C16	−179.45 (13)
C6—C7—C8—Fe1	−58.94 (10)	C15—C16—C17—C12	1.0 (2)
C7—C8—C9—C10	−0.16 (17)	C14—C13—C18—O2	−176.50 (13)
Fe1—C8—C9—C10	58.82 (11)	C12—C13—C18—O2	−0.7 (2)
C7—C8—C9—Fe1	−58.98 (11)	C14—C13—C18—O3	1.82 (19)
C7—C6—C10—C9	0.26 (17)	C12—C13—C18—O3	177.65 (12)
Fe1—C6—C10—C9	−59.72 (11)		

### 2-(Ferrocenylcarbonyl)benzoic acid (1) Hydrogen-bond geometry (Å, º)

**Table:**

*D*—H···*A*	*D*—H	H···*A*	*D*···*A*	*D*—H···*A*
O3—H3*O*···O2^i^	0.82 (2)	1.79 (2)	2.6073 (15)	174 (2)

Symmetry code: (i) −*x*, −*y*, −*z*+1.

### 3-Ferrocenyl-2-benzofuran-1(3H)-one (2) Crystal data

**Table:**

[Fe(C_5_H_5_)(C_13_H_9_O_2_)]	*F*(000) = 1312
*M**_r_* = 318.14	*D*_x_ = 1.619 Mg m^−^^3^
Monoclinic, *C*2/*c*	Mo *K*α radiation, λ = 0.71073 Å
*a* = 35.4613 (11) Å	Cell parameters from 7794 reflections
*b* = 5.6873 (2) Å	θ = 3.2–32.1°
*c* = 13.1523 (4) Å	µ = 1.16 mm^−^^1^
β = 100.2019 (16)°	*T* = 110 K
*V* = 2610.61 (15) Å^3^	Plate, yellow
*Z* = 8	0.42 × 0.38 × 0.03 mm

### 3-Ferrocenyl-2-benzofuran-1(3H)-one (2) Data collection

**Table:**

Bruker Kappa APEXII DUO CCD diffractometer	4536 independent reflections
Radiation source: fine-focus sealed tube	3996 reflections with *I* > 2σ(*I*)
TRIUMPH curved graphite monochromator	*R*_int_ = 0.028
φ and ω scans	θ_max_ = 32.1°, θ_min_ = 3.2°
Absorption correction: multi-scan (SADABS; Krause *et al.*, 2015)	*h* = −52→52
*T*_min_ = 0.826, *T*_max_ = 0.966	*k* = −7→8
18463 measured reflections	*l* = −19→19

### 3-Ferrocenyl-2-benzofuran-1(3H)-one (2) Refinement

**Table:**

Refinement on *F*^2^	Primary atom site location: structure-invariant direct methods
Least-squares matrix: full	Secondary atom site location: difference Fourier map
*R*[*F*^2^ > 2σ(*F*^2^)] = 0.047	Hydrogen site location: inferred from neighbouring sites
*wR*(*F*^2^) = 0.116	H-atom parameters constrained
*S* = 1.18	*w* = 1/[σ^2^(*F*_o_^2^) + (0.0315*P*)^2^ + 13.1297*P*] where *P* = (*F*_o_^2^ + 2*F*_c_^2^)/3
4536 reflections	(Δ/σ)_max_ = 0.001
190 parameters	Δρ_max_ = 0.91 e Å^−^^3^
0 restraints	Δρ_min_ = −0.49 e Å^−^^3^

### 3-Ferrocenyl-2-benzofuran-1(3H)-one (2) Special details

**Table:**

Geometry. All esds (except the esd in the dihedral angle between two l.s. planes) are estimated using the full covariance matrix. The cell esds are taken into account individually in the estimation of esds in distances, angles and torsion angles; correlations between esds in cell parameters are only used when they are defined by crystal symmetry. An approximate (isotropic) treatment of cell esds is used for estimating esds involving l.s. planes.

### 3-Ferrocenyl-2-benzofuran-1(3H)-one (2) Fractional atomic coordinates and isotropic or equivalent isotropic displacement parameters (Å^2^)

**Table:**

	*x*	*y*	*z*	*U*_iso_*/*U*_eq_	
Fe1	0.57389 (2)	0.67010 (6)	0.58883 (2)	0.01039 (8)	
O1	0.65076 (5)	1.0009 (3)	0.84658 (13)	0.0165 (3)	
O2	0.68358 (5)	0.9504 (4)	1.00710 (14)	0.0250 (4)	
C1	0.60472 (6)	0.8061 (4)	0.72069 (15)	0.0113 (3)	
C2	0.58955 (6)	0.5814 (4)	0.74145 (16)	0.0130 (4)	
H2	0.6048	0.4402	0.7690	0.016*	
C3	0.54919 (6)	0.5933 (5)	0.71433 (16)	0.0159 (4)	
H3	0.5310	0.4614	0.7192	0.019*	
C4	0.53896 (6)	0.8244 (5)	0.67775 (17)	0.0173 (4)	
H4	0.5123	0.8830	0.6528	0.021*	
C5	0.57335 (6)	0.9569 (4)	0.68203 (16)	0.0143 (4)	
H5	0.5751	1.1249	0.6611	0.017*	
C6	0.60854 (6)	0.6500 (4)	0.48005 (16)	0.0146 (4)	
H6	0.6358	0.7030	0.4892	0.018*	
C7	0.59580 (7)	0.4215 (4)	0.50246 (16)	0.0149 (4)	
H7	0.6125	0.2861	0.5301	0.018*	
C8	0.55499 (7)	0.4205 (4)	0.47973 (17)	0.0163 (4)	
H8	0.5380	0.2842	0.4882	0.020*	
C9	0.54272 (7)	0.6482 (5)	0.44290 (17)	0.0176 (4)	
H9	0.5155	0.6999	0.4211	0.021*	
C10	0.57580 (7)	0.7897 (4)	0.44237 (16)	0.0168 (4)	
H10	0.5760	0.9580	0.4205	0.020*	
C11	0.64568 (6)	0.8790 (4)	0.74621 (16)	0.0133 (4)	
H11	0.6521	0.9870	0.6917	0.016*	
C12	0.67423 (6)	0.6817 (4)	0.76521 (17)	0.0142 (4)	
C13	0.69187 (6)	0.6877 (4)	0.86754 (18)	0.0158 (4)	
C14	0.71987 (7)	0.5259 (5)	0.9088 (2)	0.0200 (4)	
H14	0.7316	0.5313	0.9795	0.024*	
C15	0.72993 (7)	0.3569 (5)	0.8429 (2)	0.0228 (5)	
H15	0.7491	0.2445	0.8681	0.027*	
C16	0.71201 (7)	0.3503 (5)	0.7390 (2)	0.0210 (5)	
H16	0.7192	0.2325	0.6950	0.025*	
C17	0.68418 (7)	0.5112 (4)	0.69940 (19)	0.0180 (4)	
H17	0.6722	0.5054	0.6289	0.022*	
C18	0.67642 (6)	0.8863 (4)	0.91828 (18)	0.0170 (4)	

### 3-Ferrocenyl-2-benzofuran-1(3H)-one (2) Atomic displacement parameters (Å^2^)

**Table:**

	*U*^11^	*U*^22^	*U*^33^	*U*^12^	*U*^13^	*U*^23^
Fe1	0.01266 (13)	0.01193 (14)	0.00692 (13)	−0.00146 (11)	0.00262 (9)	−0.00142 (10)
O1	0.0162 (7)	0.0157 (8)	0.0163 (7)	0.0010 (6)	−0.0007 (6)	−0.0061 (6)
O2	0.0213 (8)	0.0346 (11)	0.0175 (8)	0.0019 (8)	−0.0012 (6)	−0.0067 (8)
C1	0.0139 (8)	0.0111 (9)	0.0090 (8)	−0.0012 (7)	0.0020 (6)	−0.0018 (7)
C2	0.0148 (9)	0.0151 (9)	0.0093 (8)	−0.0028 (7)	0.0026 (7)	−0.0001 (7)
C3	0.0146 (9)	0.0228 (11)	0.0112 (9)	−0.0052 (8)	0.0045 (7)	−0.0020 (8)
C4	0.0143 (9)	0.0251 (11)	0.0128 (9)	0.0013 (8)	0.0035 (7)	−0.0049 (8)
C5	0.0177 (9)	0.0138 (9)	0.0116 (8)	0.0027 (8)	0.0031 (7)	−0.0026 (7)
C6	0.0185 (9)	0.0162 (10)	0.0109 (8)	−0.0033 (8)	0.0069 (7)	−0.0019 (7)
C7	0.0204 (10)	0.0139 (9)	0.0114 (9)	−0.0003 (8)	0.0056 (7)	−0.0015 (7)
C8	0.0209 (10)	0.0167 (10)	0.0121 (9)	−0.0051 (8)	0.0047 (7)	−0.0042 (8)
C9	0.0180 (10)	0.0241 (12)	0.0098 (8)	0.0010 (9)	0.0002 (7)	−0.0030 (8)
C10	0.0267 (11)	0.0150 (10)	0.0094 (8)	0.0001 (8)	0.0050 (8)	−0.0002 (7)
C11	0.0147 (9)	0.0121 (9)	0.0130 (8)	−0.0017 (7)	0.0020 (7)	−0.0024 (7)
C12	0.0127 (8)	0.0138 (9)	0.0168 (9)	−0.0017 (7)	0.0043 (7)	−0.0006 (8)
C13	0.0121 (8)	0.0169 (10)	0.0187 (9)	−0.0020 (8)	0.0039 (7)	−0.0006 (8)
C14	0.0132 (9)	0.0240 (12)	0.0225 (11)	0.0009 (8)	0.0025 (8)	0.0038 (9)
C15	0.0170 (10)	0.0185 (11)	0.0339 (13)	0.0043 (9)	0.0071 (9)	0.0065 (10)
C16	0.0161 (10)	0.0198 (11)	0.0280 (12)	0.0020 (9)	0.0064 (9)	−0.0008 (9)
C17	0.0166 (10)	0.0179 (11)	0.0211 (10)	−0.0011 (8)	0.0074 (8)	−0.0042 (8)
C18	0.0124 (9)	0.0198 (11)	0.0186 (10)	−0.0032 (8)	0.0021 (7)	−0.0037 (8)

### 3-Ferrocenyl-2-benzofuran-1(3H)-one (2) Geometric parameters (Å, º)

**Table:**

Fe1—C1	2.032 (2)	C6—C10	1.421 (3)
Fe1—C9	2.042 (2)	C6—C7	1.424 (3)
Fe1—C5	2.042 (2)	C6—H6	1.0000
Fe1—C8	2.045 (2)	C7—C8	1.425 (3)
Fe1—C4	2.046 (2)	C7—H7	1.0000
Fe1—C6	2.047 (2)	C8—C9	1.424 (4)
Fe1—C3	2.048 (2)	C8—H8	1.0000
Fe1—C2	2.049 (2)	C9—C10	1.423 (3)
Fe1—C7	2.051 (2)	C9—H9	1.0000
Fe1—C10	2.055 (2)	C10—H10	1.0000
O1—C18	1.356 (3)	C11—C12	1.502 (3)
O1—C11	1.474 (3)	C11—H11	1.0000
O2—C18	1.207 (3)	C12—C13	1.381 (3)
C1—C5	1.425 (3)	C12—C17	1.386 (3)
C1—C2	1.431 (3)	C13—C14	1.391 (3)
C1—C11	1.490 (3)	C13—C18	1.467 (3)
C2—C3	1.414 (3)	C14—C15	1.383 (4)
C2—H2	1.0000	C14—H14	0.9500
C3—C4	1.424 (4)	C15—C16	1.401 (4)
C3—H3	1.0000	C15—H15	0.9500
C4—C5	1.426 (3)	C16—C17	1.378 (3)
C4—H4	1.0000	C16—H16	0.9500
C5—H5	1.0000	C17—H17	0.9500
			
C1—Fe1—C9	160.95 (10)	C3—C4—H4	126.0
C1—Fe1—C5	40.95 (8)	C5—C4—H4	126.0
C9—Fe1—C5	123.48 (10)	Fe1—C4—H4	126.0
C1—Fe1—C8	157.36 (9)	C1—C5—C4	107.7 (2)
C9—Fe1—C8	40.77 (10)	C1—C5—Fe1	69.13 (12)
C5—Fe1—C8	159.82 (9)	C4—C5—Fe1	69.71 (13)
C1—Fe1—C4	68.75 (9)	C1—C5—H5	126.2
C9—Fe1—C4	106.36 (9)	C4—C5—H5	126.2
C5—Fe1—C4	40.84 (9)	Fe1—C5—H5	126.2
C8—Fe1—C4	122.84 (9)	C10—C6—C7	108.2 (2)
C1—Fe1—C6	108.60 (9)	C10—C6—Fe1	70.02 (12)
C9—Fe1—C6	68.39 (9)	C7—C6—Fe1	69.80 (12)
C5—Fe1—C6	122.32 (9)	C10—C6—H6	125.9
C8—Fe1—C6	68.51 (9)	C7—C6—H6	125.9
C4—Fe1—C6	157.29 (10)	Fe1—C6—H6	125.9
C1—Fe1—C3	68.63 (9)	C6—C7—C8	107.9 (2)
C9—Fe1—C3	120.46 (9)	C6—C7—Fe1	69.55 (12)
C5—Fe1—C3	68.64 (10)	C8—C7—Fe1	69.43 (13)
C8—Fe1—C3	106.47 (9)	C6—C7—H7	126.0
C4—Fe1—C3	40.71 (10)	C8—C7—H7	126.0
C6—Fe1—C3	161.17 (10)	Fe1—C7—H7	126.0
C1—Fe1—C2	41.07 (8)	C9—C8—C7	107.8 (2)
C9—Fe1—C2	156.00 (10)	C9—C8—Fe1	69.49 (13)
C5—Fe1—C2	68.80 (9)	C7—C8—Fe1	69.85 (13)
C8—Fe1—C2	120.98 (9)	C9—C8—H8	126.1
C4—Fe1—C2	68.41 (9)	C7—C8—H8	126.1
C6—Fe1—C2	125.37 (9)	Fe1—C8—H8	126.1
C3—Fe1—C2	40.38 (9)	C10—C9—C8	108.3 (2)
C1—Fe1—C7	122.40 (9)	C10—C9—Fe1	70.17 (13)
C9—Fe1—C7	68.44 (9)	C8—C9—Fe1	69.74 (12)
C5—Fe1—C7	158.09 (9)	C10—C9—H9	125.9
C8—Fe1—C7	40.72 (9)	C8—C9—H9	125.9
C4—Fe1—C7	159.96 (10)	Fe1—C9—H9	125.9
C6—Fe1—C7	40.65 (9)	C6—C10—C9	107.8 (2)
C3—Fe1—C7	123.88 (10)	C6—C10—Fe1	69.43 (12)
C2—Fe1—C7	108.02 (9)	C9—C10—Fe1	69.17 (12)
C1—Fe1—C10	124.89 (9)	C6—C10—H10	126.1
C9—Fe1—C10	40.66 (10)	C9—C10—H10	126.1
C5—Fe1—C10	107.68 (9)	Fe1—C10—H10	126.1
C8—Fe1—C10	68.48 (9)	O1—C11—C1	107.00 (17)
C4—Fe1—C10	121.18 (10)	O1—C11—C12	103.34 (17)
C6—Fe1—C10	40.54 (9)	C1—C11—C12	115.52 (18)
C3—Fe1—C10	156.30 (10)	O1—C11—H11	110.2
C2—Fe1—C10	162.11 (9)	C1—C11—H11	110.2
C7—Fe1—C10	68.31 (9)	C12—C11—H11	110.2
C18—O1—C11	110.93 (17)	C13—C12—C17	120.3 (2)
C5—C1—C2	108.03 (19)	C13—C12—C11	108.63 (19)
C5—C1—C11	125.5 (2)	C17—C12—C11	131.1 (2)
C2—C1—C11	126.12 (19)	C12—C13—C14	122.2 (2)
C5—C1—Fe1	69.92 (12)	C12—C13—C18	108.7 (2)
C2—C1—Fe1	70.10 (12)	C14—C13—C18	129.1 (2)
C11—C1—Fe1	130.81 (14)	C15—C14—C13	117.4 (2)
C3—C2—C1	107.9 (2)	C15—C14—H14	121.3
C3—C2—Fe1	69.79 (12)	C13—C14—H14	121.3
C1—C2—Fe1	68.83 (11)	C14—C15—C16	120.4 (2)
C3—C2—H2	126.1	C14—C15—H15	119.8
C1—C2—H2	126.1	C16—C15—H15	119.8
Fe1—C2—H2	126.1	C17—C16—C15	121.5 (2)
C2—C3—C4	108.4 (2)	C17—C16—H16	119.3
C2—C3—Fe1	69.83 (12)	C15—C16—H16	119.3
C4—C3—Fe1	69.56 (12)	C16—C17—C12	118.2 (2)
C2—C3—H3	125.8	C16—C17—H17	120.9
C4—C3—H3	125.8	C12—C17—H17	120.9
Fe1—C3—H3	125.8	O2—C18—O1	121.9 (2)
C3—C4—C5	108.0 (2)	O2—C18—C13	129.9 (2)
C3—C4—Fe1	69.73 (13)	O1—C18—C13	108.22 (19)
C5—C4—Fe1	69.45 (12)		
			
C5—C1—C2—C3	0.8 (2)	Fe1—C9—C10—C6	58.89 (15)
C11—C1—C2—C3	174.33 (19)	C8—C9—C10—Fe1	−59.56 (15)
Fe1—C1—C2—C3	−59.07 (15)	C18—O1—C11—C1	118.5 (2)
C5—C1—C2—Fe1	59.88 (14)	C18—O1—C11—C12	−3.9 (2)
C11—C1—C2—Fe1	−126.6 (2)	C5—C1—C11—O1	77.4 (2)
C1—C2—C3—C4	−0.6 (2)	C2—C1—C11—O1	−95.0 (2)
Fe1—C2—C3—C4	−59.06 (15)	Fe1—C1—C11—O1	170.79 (15)
C1—C2—C3—Fe1	58.47 (14)	C5—C1—C11—C12	−168.17 (19)
C2—C3—C4—C5	0.1 (2)	C2—C1—C11—C12	19.4 (3)
Fe1—C3—C4—C5	−59.09 (15)	Fe1—C1—C11—C12	−74.8 (3)
C2—C3—C4—Fe1	59.23 (15)	O1—C11—C12—C13	2.8 (2)
C2—C1—C5—C4	−0.7 (2)	C1—C11—C12—C13	−113.7 (2)
C11—C1—C5—C4	−174.30 (19)	O1—C11—C12—C17	−177.1 (2)
Fe1—C1—C5—C4	59.26 (15)	C1—C11—C12—C17	66.4 (3)
C2—C1—C5—Fe1	−59.99 (14)	C17—C12—C13—C14	−0.1 (3)
C11—C1—C5—Fe1	126.4 (2)	C11—C12—C13—C14	180.0 (2)
C3—C4—C5—C1	0.4 (2)	C17—C12—C13—C18	179.1 (2)
Fe1—C4—C5—C1	−58.90 (14)	C11—C12—C13—C18	−0.8 (2)
C3—C4—C5—Fe1	59.27 (15)	C12—C13—C14—C15	0.5 (4)
C10—C6—C7—C8	−0.7 (2)	C18—C13—C14—C15	−178.5 (2)
Fe1—C6—C7—C8	59.02 (15)	C13—C14—C15—C16	−0.6 (4)
C10—C6—C7—Fe1	−59.70 (15)	C14—C15—C16—C17	0.3 (4)
C6—C7—C8—C9	0.3 (2)	C15—C16—C17—C12	0.1 (4)
Fe1—C7—C8—C9	59.36 (15)	C13—C12—C17—C16	−0.2 (3)
C6—C7—C8—Fe1	−59.09 (15)	C11—C12—C17—C16	179.7 (2)
C7—C8—C9—C10	0.2 (2)	C11—O1—C18—O2	−176.6 (2)
Fe1—C8—C9—C10	59.83 (15)	C11—O1—C18—C13	3.6 (2)
C7—C8—C9—Fe1	−59.58 (15)	C12—C13—C18—O2	178.5 (3)
C7—C6—C10—C9	0.8 (2)	C14—C13—C18—O2	−2.3 (4)
Fe1—C6—C10—C9	−58.73 (15)	C12—C13—C18—O1	−1.7 (3)
C7—C6—C10—Fe1	59.56 (14)	C14—C13—C18—O1	177.5 (2)
C8—C9—C10—C6	−0.7 (2)		

### 3-Ferrocenyl-2-benzofuran-1(3H)-one (2) Hydrogen-bond geometry (Å, º)

**Table:**

*D*—H···*A*	*D*—H	H···*A*	*D*···*A*	*D*—H···*A*
C6—H6···O2^i^	1.00	2.58	3.470 (3)	148

Symmetry code: (i) *x*, −*y*+2, *z*−1/2.
